# Mitochondrial Lipid Homeostasis at the Crossroads of Liver and Heart Diseases

**DOI:** 10.3390/ijms22136949

**Published:** 2021-06-28

**Authors:** Siarhei A. Dabravolski, Evgeny E. Bezsonov, Mirza S. Baig, Tatyana V. Popkova, Alexander N. Orekhov

**Affiliations:** 1 Department of Clinical Diagnostics, Vitebsk State Academy of Veterinary Medicine (UO VGAVM), 7/11 Dovatora Street, 210026 Vitebsk, Belarus; 2 Laboratory of Cellular and Molecular Pathology of Cardiovascular System, Institute of Human Morphology, 3 Tsyurupa Street, 117418 Moscow, Russia; evgeny.bezsonov@gmail.com (E.E.B.); a.h.opexob@gmail.com (A.N.O.); 3 Laboratory of Angiopathology, The Institute of General Pathology and Pathophysiology, 8 Baltiyskaya Street, 125315 Moscow, Russia; 4 Department of Biosciences and Biomedical Engineering (BSBE), Indian Institute of Technology Indore (IITI), Simrol 453552, India; msb.iit@iiti.ac.in; 5 V.A. Nasonova Institute of Rheumatology, 34A Kashirskoye Shosse, 115522 Moscow, Russia; popkovatv@mail.ru

**Keywords:** NAFLD, cardiovascular disease, dyslipidemia, atherosclerosis, fatty-acid β-oxidation, lipid metabolism

## Abstract

The prevalence of NAFLD (non-alcoholic fatty liver disease) is a rapidly increasing problem, affecting a huge population around the globe. However, CVDs (cardiovascular diseases) are the most common cause of mortality in NAFLD patients. Atherogenic dyslipidemia, characterized by plasma hypertriglyceridemia, increased small dense LDL (low-density lipoprotein) particles, and decreased HDL-C (high-density lipoprotein cholesterol) levels, is often observed in NAFLD patients. In this review, we summarize recent genetic evidence, proving the diverse nature of metabolic pathways involved in NAFLD pathogenesis. Analysis of available genetic data suggests that the altered operation of fatty-acid β-oxidation in liver mitochondria is the key process, connecting NAFLD-mediated dyslipidemia and elevated CVD risk. In addition, we discuss several NAFLD-associated genes with documented anti-atherosclerotic or cardioprotective effects, and current pharmaceutical strategies focused on both NAFLD treatment and reduction of CVD risk.

## 1. Introduction

The prevalence of NAFLD is a rapidly increasing problem, affecting 25–45% of the adult population worldwide and up to 70% in T2DM (type 2 diabetes mellitus) and obesity patient groups [1]. It is known that NAFLD is associated with many co-morbidities (such as hypertension, obesity, MetS (metabolic syndrome) and hyperlipidemia). NASH (non-alcoholic steatohepatitis) a more severe form, affects 2–7% of adults and could further progress to cirrhosis form or HCC (hepatocellular carcinoma). Despite the liver-related complication, CVD is a common cause of death among NAFLD patients [2]. However, it is important to note, while most probably all types of NAFLD are associated with elevated CVD risk, the strongest links were defined for NASH and advanced stages of fibrosis [3,4].

The exact mechanism responsible for the connection between NAFLD and CVD is not proven. However, during recent years, several “drivers” were proposed to be responsible for NAFLD progression and accelerating atherogenesis: dyslipidemia, chronic inflammation, and endothelial dysfunction [5]. The accompanying expansion of adipose tissue initiates a pro-inflammatory cascade with the NF-kB (nuclear factor kappa B) and JNK (c-Jun N-terminal kinase) pathways. Further NAFLD complications may include IR (insulin resistance) (hepatic or system-wide level), increased production of inflammatory cytokines (IL-6 (Interleukin 6), C-reactive protein, TNF-α (tumour necrosis factor-alpha), and others), synthesis of procoagulant factors (factor VIII, endothelin, TGF-β (transforming growth factor-beta), fibrinogen, and others) and hepatokines, dysregulated glucose, and lipid metabolism [6,7].

Atherosclerotic neo-intimal plaques develop in large arteries and drive adverse CV events (such as stroke and myocardial infarction). Atherosclerosis is a decades-lasting chronic disease, during which inflammation, calcification, fibrosis, and lipid-deposition change the composition of atherosclerotic plaques [8]. Several methods have been used to detect plaque features and evaluate CVD risk: invasive, used mostly on more advanced stages (angiography, optical coherence tomography, or intravascular ultrasonography); and non-invasive, more often used for initial diagnostics (positron emission and computer tomography, measurement of carotid intima-media thickness, and others) (reviewed in [9]).

Results of many cross-sectional studies, meta-analyses and systematic reviews suggest that NAFLD increases the risk of atherosclerosis and favours the development of unstable plaques [10,11,12,13]. In addition, genetic evidence suggests that NAFLD-mediated dyslipidemia is a crucial factor of elevated CVD risk [14]. While many genetic polymorphism sites and mutations are associated with both CVD [15,16] and NAFLD [17], some NAFLD favouring SNPs (single-nucleotide polymorphisms) have been described as decreasing CVD risk [18,19,20]. Other research, however, found no such protection [21,22]. For readers interested in immuno-inflammatory aspects and the role of bile acid and cholesterol metabolism in NAFLD–CVD relations, we recommend recent excellent reviews [23,24,25,26,27].

### Association between Liver and Heart Disease

Several mechanisms that explain the close connection between CVD and NAFLD have been suggested. It is known that IR plays a crucial role in NAFLD and NASH pathogenesis [28]. In addition, IR affects many physiological processes and causes hyperglycemia and dyslipidemia, activating low-grade chronic inflammation, ectopic lipid accumulation, OS (oxidative stress), and endothelial dysfunction [29]. An elevated level of serum ferritin, the main iron-storing protein, also is common for NAFLD patients, and is associated with IR [30]. Altogether, these events create a so-called CVD-favouring pro-atherogenic environment [31]. Combined with altered immune-cell populations, defined in NASH patients [23], this suggests an immune and chronic inflammatory link between IR, CVD, and DM [32]. Fetuin-A, a glycoprotein secreted from adipose tissue and liver, stimulates the production of inflammatory cytokines from adipocytes and macrophages, and serves as a biomarker of several chronic inflammatory diseases. The level of fetuin-A (fatty-acid carrier) also is increased in NAFLD/NASH patients [33]. It was recently shown that fetuin-A could inhibit insulin receptor tyrosine kinase in muscle and liver and cause IR [34]. The level of fetuin-A was linked to hypertriglyceridemia, and there was no significant association with risk of ischaemic stroke and other CVD [35], while other research suggests it as a valuable factor for chronic heart failure diagnostics [36].

Different extents of dyslipidemia often present in NAFLD/NASH patients (decreased level of HDL-C, and increased levels of LDL particles and TG (triglycerides)), and serve as an important non-invasive marker for NAFLD diagnostics [27,37]. However, such a lipid profile is known as atherogenic and was also linked with the severity of cardiometabolic risk [38,39]. The liver is the central organ responsible for lipid metabolism, among which cholesterol and TG are of the most importance. While low HDL-C is the well-defined marker for NAFLD and a risk factor for CVD, the exact molecular mechanism responsible for this connection is under intensive investigation [40]. One of the best-known functions of HDL is the ability to promote RCT (reverse cholesterol transport), which allows removal of excess cholesterol from the macrophages with further excretion from the body with bile. RCT has attracted much attention as a promising therapeutic target to reduce CVD risk [41]. However, therapeutic improvement of HDL-C level with drugs was not beneficial to lower CVD risk, thus suggesting a more complex relationship between HDL-C and CVD [42].

An elevated level of serum homocysteine is a well-known cause of hepatic oxidative stress and hepatic steatosis [43], correlating with the level of liver dysfunction in NAFLD/NASH patients [44]. Similarly, serum homocysteine serves as an independent factor for CVD [45]. Homocysteine is known to activate Toll-like receptor 4, dysregulate Ca^2+^ and NO signalling, increase production of ROS, and induce platelet activation and endothelial dysfunction, which eventually cause CVD [46].

Another connection point between NAFLD and CV effects is inflammatory cytokines, which are released by the liver, and cause system inflammation and promote CVD. The main inflammation-mediated triggers leading to CVD are enhanced plaque formation, alteration in vascular tone, coagulation, and endothelial function [47]. Levels of several cytokines (such as IL-1, IL-6, C-reactive protein, and TNFα) known as system inflammation markers are elevated in NAFLD patients [48]. Recent research suggests an association of liver steatosis and fibrosis with diastolic heart dysfunction and impaired myocardial glucose uptake [49]. In addition, hepatic fat content was linked to increased left ventricular filling pressure, which is a precursor of heart failure [50].

Future research should be concentrated on the early detection of the metabolic markers of liver and heart efficiency, ideally before functional and structural abnormalities appear. Thus, individuals with diagnosed NAFLD would have a possibility to prevent complications with the CV system. In this review, we focus on the role of liver lipid homeostasis and mitochondrial β-oxidation in the connection between NAFLD and CVD, associated genetic regulations, and targeted therapies.

## 2. Liver as a Central Organ for Lipid Metabolism

The liver plays a crucial role in lipid and glucose homeostasis and metabolism. Under the pressure of continuous impaired FA (fatty acid) metabolism, the liver accumulates a significant amount of lipids that lead to NAFLD development. The main feature of NAFLD is an accumulation of hepatic TGs, which could be caused by internal (impaired FAO, VLDL synthesis and export), external (certain genetic background and environmental conditions) or behavioural (exceed FAs from the diet, circulation or adipose tissue, lack of physical activities) triggers [51,52]. An imbalance between imported/exported and synthesized/processed FAs results in hepatic lipid accumulation, hepatosteatosis and IR, which is further stimulating *de novo* hepatic lipogenesis making this vicious cycle complete [53]. While the exact molecular mechanisms are not completely understood and are under intensive investigation, impaired FAs metabolism and ROS production, leading to chronic inflammation and mitochondria malfunction have been suggested as key events in this disease development [54,55].

### 2.1. Lipids Homeostasis in the Liver Mitochondria: Fatty-Acid β-Oxidation

**Figure 1:**
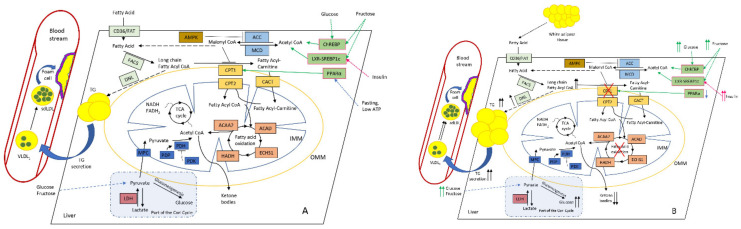
Altered hepatic lipid metabolism in normal (**A**) and NAFLD (**B**) conditions. Increased diet supply of glucose and fructose affects ChREBP (carbohydrate-responsive element-binding protein), SREBP1c (sterol regulatory element-binding protein-1c), and LXR (liver X receptor) TFs, which stimulate malonyl CoA synthesis. PPARα (peroxisome proliferator-activated receptor alpha) normally activates CPT1 under fasting and low-ATP conditions [58]. As a key intermediate, malonyl CoA inhibits CPT1, thus reducing FAO. This leads to the accumulation of long-chain fatty-acid CoA (which could also be delivered from surplus adipose tissue), and stimulates DNL with the subsequent rise in intrahepatic TG and plasma TG levels, further increasing large VLDL_1_ and the formation of small dense LDL, which favours foam cell formation and ultimately atherosclerosis. During the progression of NAFLD, the production of ketone bodies progressively reduces while hepatic glucose synthesis and output increases, thus further promoting IR and the rise in insulin level [59]. Colour coding as follows: PPARα and other TFs are depicted in pale green; primarily FA-metabolising enzymes (FAT (fatty-acid translocase) and FACS (fatty-acid synthase)) and DNL (de novo lipogenesis) are highlighted in pale blue; malonyl-CoA-metabolising enzymes ACC (acetyl-CoA carboxylase) and MCD (malonyl-CoA decarboxylase) are highlighted in blue; CPT system enzymes (CPT1, CPT2, and CACT) are depicted in light brown; AMPK (AMP-activated protein kinase), the main regulator of CPT system are depicted in brown; FAO enzymes ACAA2 (acetyl-CoA acyltransferase 2), ACAD (acyl-CoA dehydrogenase), ECHS1 (enoyl-CoA hydratase, short chain 1), and HADH (hydroxyacyl-CoA dehydrogenase) are depicted in in pale red; pyruvate metabolism enzymes MPC (mitochondrial pyruvate carrier 1), PDP (pyruvate dehydrogenase phosphatase), PDH (pyruvate dehydrogenase), and PDK (pyruvate dehydrogenase kinase) are depicted in dark blue; and LDH (lactate dehydrogenase) is depicted in red. Abbreviations: IMM and OMM, inner and outer mitochondrial membrane, respectively.

Carbohydrates and FAs are the main energy source for cells, and their uptake from the extracellular space and intracellular release is tightly controlled by several hormones, such as glucagon, insulin, noradrenaline, and others. Inside the cell, FAs are esterified, metabolized to the lipid second messengers (such as ceramide, sphingosine, phosphatidylinositol bisphosphate, and others), or transport to the mitochondria for β-oxidation. However, a VLCFA (very-long-chain fatty acid) (FAs with 22 and more carbons) could not be metabolized in the mitochondria, and should be delivered to peroxisomes (reviewed in [56]). After LCFAs (long-chain FAs) are activated by CoA, LCFA-CoA ester is transported into the mitochondrial matrix via the CPT (carnitine palmitoyltransferase) system, which consists of three proteins: CPT1, CPT2, and CACT (acylcarnitine translocase) [57] (Figure 1).

CPT1 could be inhibited by malonyl-CoA derived from glucose metabolism, thus making CPT1 the rate-limiting step in mitochondrial FAO. There are three isoforms of CPT1, which are organ-specific for liver (A), muscle and heart (B), and brain (C). Two more enzymes are involved in the malonyl-CoA metabolism: ACC (acetyl-CoA carboxylase) is responsible for the synthesis of malonyl-CoA, and MCD (malonyl-CoA decarboxylase) is responsible for the degradation of malonyl-CoA [60].

There are two main ACC isoforms, ACC1 and ACC2, which have different tissue expression patterns and functions. ACC1 is localized in the cytoplasm of all cells, but is enriched in lipogenic tissue (such as adipose tissue) [61]. ACC2 is localized in the mitochondria and enriched in oxidative tissue (such as heart and skeletal muscle) [62]. Thus, different tissues have a specific ACC1/ACC2 ratio, which is required to balance FA oxidation and synthesis. ACC1 and ACC2 are both highly expressed in the liver, where processes of both FA oxidation and synthesis are important. However, such a difference in ACC localization and function provides an opportunity to create pharmaceutical drugs for specific inhibition of FA synthesis and stimulation of fatty-acid oxidation, which could be beneficial for some morbidities such as obesity, NAFLD, diabetes, and others [63,64].

AMPK is one of the main regulators of this pathway; it acts via phosphorylation and inhibition of ACCs, thus decreasing expression of FA synthase and supply of intermediates for the FA anabolic pathway. As a secondary and long-term effect, AMPK phosphorylates TFs SREBP1c and ChREBP, thus inhibiting the transcription of subsequent lipogenic genes [65]. Sirtuin proteins (SIRT1 and SIRT3) stimulate AMPK via deacetylation of its upstream activator LKB1 (liver kinase B1) [66]. Nowadays, sirtuins are recognised as crucial regulators of lipid metabolism, providing tissue-specific FAO-promoting activities (in skeletal muscle and liver), lipolysis (in adipose tissue), mitochondrial respiration (BAT (brown adipose tissue)), and food intake (in the hypothalamus) [67].

Mitochondrial FAO in the liver leads to complete oxidation to CO_2_, or partial when ketone bodies, an exported form of energy-containing molecules, are formed. The data regarding the CPT1A levels of expression and activity and rate of mitochondrial FAO are controversial and greatly depend on the model system used, FFA concentration, and other experimental conditions [68,69]. To explain these differences, several mechanisms have been suggested: (1) the levels of malonyl-CoA are variable and depend on the ratio of ACC/MCD proteins; (2) the physical properties of the mitochondrial membrane could change the sensitivity of CPT1A to malonyl-CoA [70]; and (3) the available pool of FFAs and other lipid intermediates could activate different transcription factors responsible for FA de novo synthesis, uptake, transport, and oxidation [71,72].

The current model explains the relationship between FFAs and FAO as the hormetic effect when a mild or evanescent rise in available FFAs leads to beneficial increased FAO with higher energy output. However, prolonged and significant overflow of FFAs leads to an excessive electron flux in the ETC (electron transport chain), ROS overproduction, and formation of toxic aldehydes, which damage mitochondrial proteins, lipids, and DNA, and cause morphological and functional disturbances [73,74,75]. As a possible strategy to avoid such harmful effects, the liver can switch the balance from complete FA oxidation towards ketone-body production [76]. However, similar to the FAO pathway, this strategy has its limits, and could cause some further complications under prolonged FFA overflow and reduced energy expenditure [77,78].

### 2.2. Interplay and Co-Regulation with Glucose Metabolism

The modern diet (especially of typical Western style) contains a high amount of simple fructose- and glucose-based saccharides, which are risk factors for the development of several metabolic complications, such as obesity, T2DM, NAFLD, CVD, and others [79,80]. Linoleic acid is a polyunsaturated omega-6 fatty acid, also widely presented in the Western diet and associated with weight gain, obesity, IR, and CVD [81,82,83]. It is known that fructose serves as a substrate for FA synthesis and stimulates the TFs of de novo lipogenesis and triglyceride synthesis, SREBP1c and ChREBP [84]. Simultaneously, fructose decreases FAO via two main mechanisms: increasing the level of hepatic malonyl-CoA, and directly altering the expression of hepatic genes responsible for lipid accumulation and removal [85]. The unique aspect of fructose action is a transient decrease in intracellular levels of phosphate and ATP, which is associated with the uric acid generation and nucleotide turnover. A decreased ATP level induces a series of reactions, including induction of OS, a transient block in protein synthesis, and mitochondrial dysfunction, which turned out to have a key role in fructose-mediated effects [86,87]. Because different CPT1 isoforms have different sensitivities to malonyl-CoA, where liver isoform is 30–100 times less sensitive in comparison to heart and muscle isoforms [88,89], fructose’s malonyl-CoA-independent effect on liver mitochondrial FAO may be more pronounced and promote a higher degree of mitochondrial dysfunction. Additionally, diet-delivered nutrients could regulate mitochondrial functions via post-translational modifications (malonylation, acetylation, succinylation, and others) [90,91,92]. However, the discussion of these modifications is beyond the scope of this manuscript, and we wish to redirect interested readers to the cited papers.

### 2.3. Role of Perilipin 5 in NAFLD and Atherosclerosis

Plin5 (perilipin 5) is an important member of the perilipin protein family, which is abundant in tissues with very active lipid catabolism, such as the heart, skeletal muscle, brown adipose tissue, and the liver [93]. NAFLD is characterized by increased accumulation of LDs in the liver, as well as increased expression of PLIN5 [94]. Plin5 is known as the main LD forming and coating protein, responsible for restoring hepatic TGs in LDs and inhibition of lipolysis. Not surprisingly, Plin5 overexpression worsens hepatosteatosis [95], and simultaneously blocks stellate-cell activation [96,97], but without adverse effects on IR. On the other hand, PLIN5 deficiency leads to impaired insulin signal transduction and the development of IR [98].

Recent studies have suggested the molecular mechanism explaining these observations. The C-terminal part of the Plin5 (443–63aa) recruits mitochondria to contact LDs. Such LD–mitochondria contact is required for proper supply of FAs to mitochondria, lipid synthesis, and LD expansion [99]. These features refer to the role of mitochondria in the synthesis of TAGs and phospholipid, because enzymes, localized on the outer membrane of mitochondria GPAT1 and 2 (glycerol-3-phosphate acyltransferase 1 and 2) and AGPAT (1-acyl glycerol-3-phosphate acyltransferase) are responsible for the biosynthesis of lysophosphatidic acid and phosphatidic acid, respectively [100]. Mitochondria, associated with LDs, have increased capacities for pyruvate oxidation, electron transport, and ATP synthesis, a reduced β-oxidation capacity, and uniquely low fusion–fission dynamics [101]. Further, Plin5 was shown to limit FA toxicity, clear harmful proteins from the outer mitochondria membrane, and protect against OS [102].

PLIN5 showed implications in the inflammatory response via activation of the NLRP3 (NLR family pyrin domain-containing 3) inflammasome, thus linking it to the NAFLD-to-NASH progression [103]. In addition, a high level of Plin5 was found in HCC biopsy specimens [104]. However, the data regarding PLIN5′s role in HCC development and metastasis are still limited, and further research is required to elucidate the exact function of PLIN5 in HCC.

Interestingly, PLIN5 deficiency was also implicated in the progression of atherosclerosis, as was shown in double knockout mice (ApoE^−/−^Plin5^−/−^) that developed more severe atherogenesis (with elevated TG, TC, and LDL-C levels, and reduced HDL-C contents) and accelerated inflammation, apoptosis, lipid accumulation, and OS. Mutant mice had promoted atherogenesis progression, along with an increased entire aorta, aortic arch, and abdominal aorta area [105]. Additionally, PLIN5 is involved in thermoregulation and adaptation to cold stress. A study of LDs and mitochondria isolated from the liver of mice housed at chronic cold stress determined that the mitochondrial TCA cycle and retinol metabolism were enhanced, while oxidative phosphorylation was not affected. Liver adaptation to cold stress conditions involved increased expression of Plin5 and MUPs (major urinary proteins), whereas expression of MPC was dramatically decreased [106]. Previously, the role of thermogenesis in the development of metabolic diseases was associated mostly with adipose tissues (interplay and transition between both white and brown) [107,108]. However, the described cold-adaptation-related role of Plin5 also implies a role of the homeostasis and thermogenesis of liver lipids in NAFLD development [106].

These findings elucidate that PLIN5 is a crucial pleiotropic regulator of hepatic lipid metabolism, thermogenesis, and inflammatory response involved in NAFLD/NASH and atherosclerosis development and progression. PLIN5 is a promising therapeutic target for NAFLD and atherosclerosis, and possibly for some other metabolic diseases. We wish to redirect interested readers to recent reviews for further information about the role of PLIN5 and mitochondria-LD contact sites in the development of NAFLD and other diseases [109,110].

### 2.4. Role of the Liver Mitochondria in the Development of CVD-Promoting Dyslipidemia

A certain degree of atherogenic dyslipidaemia is present in NAFLD/NASH patients that is characterized by TC/HDL-C, LDL-C/HDL-C, and TG/HDL-C ratios [111,112,113]. Due to the known central role of the liver in the production/clearance of all classes of lipoprotein (HDL) and apolipoproteins (ApoB48 and ApoB100), it makes a solid connection between NAFLD/NASH-associated metabolic dysfunction and elevated CVD risk [114]. In this section, we focus on recent mechanistic insights into links between genes known to protect/cause NAFLD, ameliorate/worsen the NAFLD phenotype, alter liver-specific lipid accumulation, or influence the lipid profile at a system-wide level (Table 1).

In general, we could distinguish several groups of genes. The first one combines genes necessary to support mitochondrial functionality (mitochondrial DNA replication, protein synthesis, fission/fusion, integrity, ROS, and bioenergy production). For the second group, we categorized genes participating in fatty-acid β-oxidation (enzymes providing biochemical output and several regulators). The third group has antioxidant genes (*SOD*, *PRX5*, and *CAT*) with known lipid-metabolism and fat-accumulation phenotypes. The next group contains different genes that participate in general lipid metabolism (de novo biosynthesis, uptake, and secretion). The “Supplementation” group combines several diet-intervention studies in which different treatments were used to define their effect on the lipid profile and FAO. The final group contains several genes that participate in the different stages of liver lipid metabolism. Such grouping was very conditional and was done only for the convenience of our discussion. There are many genes implicated in FAO disorders that are not listed here due to the absence of the NAFLD/NASH-related phenotype; however, these genes are relevant for this topic, and we wish to redirect interested readers to the recent study [178].

Analysis of described mutations suggested the central place of liver mitochondrial FAO in the connection between NAFLD and CVD risk. The key points were: (1) increased FAO may alleviate NAFLD symptoms, but cause hypertriglyceridemia and OS damage to the liver; (2) proper antioxidant supply may support FAO by neutralizing excess ROS; (3) complete absence of FAO in liver mitochondria causes resistance to HFD obesity and NAFLD, along with serum dyslipidemia and hepatic OS; (4) proper mitochondria turnover may support effective FAO and ameliorate NAFLD symptoms; (5) complete absence/reduction of FAO enhanced energy expenditure at the system-wide level and suppressed adiposity; (6) with disabled FAO, other mechanisms of FA oxidation are stimulated in other compartments/cells (peroxisomes and microsomes, macrophages, and others). Thus, analysis of collected mutations supported the hypothesis of a hormetic effect [74] when the evanescent rise of input FFAs is beneficial and stimulates FAO, providing higher energy output. Excess or prolonged stimulation of FAO is harmful; the depleted antioxidant pool could not cope with increased ROS production, which caused mitochondrial and liver damage, as well as dyslipidemia with further complications to the CV system. There are several possible pathways that could be responsible for such effects. ROS overproduction may cause lipid peroxidation, which forms a 4-hydroxynonenal-CPT1 adduct, responsible for impaired FAO and lipid removal from hepatocytes. In addition, ROS attack PUFAs, thus initiating lipid peroxidation and the formation of toxic aldehyde by-products (a hydroxy-2-nonenal and malondialdehyde), which have longer half-lives than ROS, and can spread from their site of origin to reach distant intracellular and extracellular targets, thereby amplifying the effects of OS [179].

Under normal physiologic conditions, oxidation of long- and medium-chain FAs is primarily run by the mitochondrial β-oxidation system, with only a minor contribution from the peroxisomal system. VLFAs (very-long-chain fatty acids) are not substrates of CPT-1, and thus cannot enter mitochondria; however, VLFAs are preferential substrates for peroxisomal β-oxidation [180]. Despite the presence in the peroxisome of the full enzymatic machinery to β-oxidize FAs, such oxidation normally is incomplete, and the final products of the peroxisomal β-oxidation are shuttled to mitochondria for complete oxidation to CO_2_, H_2_O, and final energy output. Some products of peroxisomal β-oxidation also may be used in other metabolic pathways (for example, by participating in the biosynthesis of the taurine and glycine conjugates, with subsequent export into the biliary ducts) [181].

FAs could also be subjected to ω-oxidation by the action of a microsomal oxidase that uses molecular oxygen, and both an alcohol and aldehyde dehydrogenase to produce dicarboxylic acids. These dicarboxylic acids can be further degraded by peroxisomal β-oxidation to succinate and acetyl-CoA, or completely oxidized after transport into the mitochondrial β-oxidation system [182]. Under physiologic conditions, ω-oxidation is a minor pathway of FA metabolism, but a failure of β-oxidation can result in increased ω-oxidation activity, with a production of excess dicarboxylic acids that are non-specific markers of mitochondrial FAO defects [183].

In the case of complete loss of mitochondrial FAO, the increased levels of serum hepatokines were detected (IGFBP1 (insulin-like growth factor-binding protein 1), GDF15 (growth/differentiation factor 15), and FGF21), suggesting their role in physiological adaptations to the high lipid burden from an HFD [73]. The liver attempts to compensate for the loss of FAO by up-regulating oxidative programming and seeking to increase catabolism in peripheral tissues with help of secreted hepatokines [184]. The primary target for those hepatokines is enhanced energy expenditure from adipose tissue (brown) and stimulation of browning (for white) [185]. Additionally, macrophages are also known to participate in BAT thermogenesis [186].

The important role of macrophages was also supported by recent research, in which macrophage FAO was defined as athero-protective, while inhibition of macrophage FAO may increase foam-cell formation and thereby exacerbate atherosclerosis [187]. Interestingly, FAO inhibition leads to increased ROS levels, probably due to the accumulation of toxic partially metabolized FAO substrates [188]. In this light, NAFLD-associated genes with documented anti-atherosclerotic or cardioprotective effects are intriguing, and we now further discuss some known cases for both metabolic and genetic NAFLD.

## 3. Cardioprotection

GCN2 (general control nonderepressible 2) is an amino-acid-availability sensor, identified in many organisms. In mammals, the highest level of GCN2 expression was detected in the liver and brain. Limited dietary proteins intake activates GCN2, which phosphorylates eIF2α (eukaryotic initiation factor 2 alpha) to inhibit global protein translation and stimulate de novo amino acid biosynthesis to restore homeostasis [189]. Further downstream targets are ATF4 (activating transcription factor 4) and CHOP (C/EBP homologous protein), which up-regulate autophagy and biosynthesis pathways [190]. Recently it was shown that GCN2 also participates in the regulation of hepatic lipid metabolism. For example, GCN2 deficiency significantly attenuated HFD-induced liver dysfunction, hepatic steatosis, and IR via regulation of lipogenic genes (*SREBP-1*/*PPARγ*) and their downstream targets (FASN, CD36, SCD1) [141]. Other research, focused on the combined effect of the GCN2 deficiency and exercise on hepatic steatosis, also defined involvement of the AMPK/SIRT1/PPARα pathway [142].

Interestingly, GCN2 deficiency was also shown to have a cardioprotective effect in diabetic hearts. In particular, GCN2 knockdown reduces OS, cell death, and lipid accumulation via inhibiting eIF2α-ATF4-CHOP signalling with the following reduction of Bcl-2/Bax ratio and UCP2 (uncoupling protein 2) expression [143,144]. Thus, GCN2 target therapy may be a promising strategy in the case of diabetic cardiomyopathy, and also as a treatment to reduce cardiotoxic side effects of popular anti-cancer drugs such as Doxorubicin.

Recent research helped to establish different mechanisms of the CVD risk in the case of NAFLD sub-types (metabolic and genetic). It is known that carriers of many SNP sites and mitochondrial mutations have a higher susceptibility to NAFLD [17]. However, a protective effect against CAD (coronary artery disease) was shown for several such SNPs [18], suggesting that every mutation site could imply a unique mechanism of NAFLD susceptibility/CVD protection [19]. Indeed, recent meta-analysis research showed that NAFLD susceptibility genes do not cause CAD per se [191]. Among NAFLD SNPs carriers, a strong correlation was observed for TC (total cholesterol) and LDL-C with CAD, but not for plasma TG and HDL-C. Overflow of FAs and de novo lipogenesis initiate fat accumulation in the liver and drive further VLDL production, thus shifting the plasma lipid balance into a pro-atherogenic and CVD-favouring environment [40]. However, some NAFLD-associated SNPs have impaired VLDL secretion (TM6SF2 and PNPLA3 (patatin-like phospholipase domain containing 3), and also MTTP and PEMT), thus decreasing plasma lipids and providing a cardioprotective effect [191].

Currently, PNPLA3 I148M is one of the best-studied NAFLD-associated mutations [192]. By itself, the Pnpla3148M variant is not harmful, and experimental mice on a standard diet had a normal level of liver fat [193]. However, the level of liver fat was 2–3 times higher under a high-sucrose diet, with an approximately 40-times-higher level of PNPLA3 protein on hepatic LDs, while Pnpla3 mRNA level was not changed [193]. Further, it was shown that a mutant variant could escape ubiquitination and proteasomal degradation, leading to such significant protein accumulation on LDs [194]. Normally, PNPLA3 acts as a PUFA-specific lipase or transacylase, yielding PUFA-containing PCs (phosphatidylcholines) or DAGs (diacylglycerols) that could be used for PC synthesis [195]. It is important to note that the Pnpla3148M variant and PNPLA3 deficiency had similar effects on liver lipid metabolism, thus preventing TG mobilization and causing lipid accumulation in hepatocytes. In certain conditions it could cause NAFLD development; however, for the cardiovascular system, such TG retention in the liver has a positive system-wide effect on the serum lipids profile, resulting in a lower risk of CVD [196].

TM6SF2 (transmembrane 6 superfamily member 2) is a transmembrane protein localized mainly in the ER and Golgi of enterocytes and hepatocytes. E167K mutations significantly reduce TM6SF2 protein levels, causing high hepatic accumulation of TG but low plasma level of LDL-C, and are thus responsible for a NALFD susceptibility and CVD protection, respectively [191]. TM6SF2 deficiency mimics the NAFLD phenotype, while liver-specific *TM6SF2* OE elevates plasma TC and LDL-C levels [197]. ERLIN (ER lipid raft protein 1 and 2) proteins are ER-localized transmembrane glycoproteins that participate in the regulation of the cholesterol biosynthetic pathway by blocking the export of SREBPs from the ER to the Golgi under high-cholesterol conditions [198]. Recent research has found that TM6SF2 could bind and stabilize both ERLINs and APOB, therefore serving as a connective hub between ERLINs and APOB. E167K mutation in TM6SF2, equal to ERLIN or TM6SF2 deficiencies, leads to defective APOB stabilization, which is one of the key factors in the development of this sub-type of genetic NAFLD [150].

In total, these studies highlight the fundamental difference between metabolic and genetic NAFLD. Thus, for NAFLD patients carrying specific SNP sites, several independent molecular pathways could be involved in quicker NAFLD progression, but also accompanied by some extent of cardioprotection. In such cases, personalized, genotype-based medicine should be applied, based on presented SNP site/s, the severity of other symptoms, and the presence of other co-morbidities.

## 4. Pharmaceutical Strategies to Treat NAFLD and Reduce CVD Risk

The primary lifestyle interventions recommended for NAFLD patients are diet modifications and physical exercise. The most effective diet modification is a low-carbohydrate, ketogenic, low-fat and Mediterranean diet, which provides a positive effect on dyslipidemia, hepatic steatosis, and related comorbidities [76,199]. Similarly, different physical activities (high-intensity interval, aerobic, and resistance training) have been shown to reduce liver fat content and body weight, and improve plasma lipid status and IR. In addition, such exercises were followed by improvements in CVD risk factors, such as plasma levels of TG-rich VLDL_1_ particles and LDL-C, and reduced arterial stiffness [200]. Another type of effective NAFLD and NASH treatment, especially when accompanied with severe obesity, is bariatric surgery, which is aimed to mechanically reduce food intake [201]. Bariatric surgery also reduced the risk of CVD events among T2D and obese patients; thus, a similar effect would be also expected in the case of NASH patients [202].

Currently, there are no approved pharmacological therapies for NAFLD/NASH treatment. Existing treatments aim to reduce liver fat accumulation, stimulate metabolic pathways, and decrease liver injury. The main classes of such medications are: (1) bile acid metabolism modulators; (2) PPAR agonists; (3) thyroid hormone receptor β agonists; and (4) well-known T2DM drugs (such as GLP1-targeted drugs) [203]. Further, we focus on the FGF21 analogues, one of the most promising drugs for NAFLD/NASH treatment with documented effect on lowering CVD risk.

FGF21 (fibroblast growth factor 21) is an endocrine hormone of the FGF family, which is secreted mainly by the liver and exhibits diverse metabolic activities. The FGF21 signalling pathway begins with binding the co-receptors KLB (β-Klotho) and FGFR1 (FGF receptor 1), which forms an active FGF21/FGFR1/KLB receptor complex. Such a triple complex can phosphorylate ERK1/2 (extracellular signal-regulated kinases 1 and 2) and FRS2α (fibroblast growth factor receptor substrate 2 alpha) with multiple downstream targets [204]. The exact signalling cascade of FGF21 has not been fully resolved; it is known that in the liver, FGF21 expression is regulated by PPARα and could be also repressed by LXR [205,206]. FGF21 plays an important role in glucose and lipid metabolism, and insulin sensitivity. FGF21 is produced primarily by the liver in response to metabolic stresses, such as ketogenic diet or fasting, and is required for regulation of lipolysis, ketogenesis, and FAO [207]. Elevated levels of FGF21 (both circulation and mRNA) have been observed in cases of several metabolic disorders (NAFLD, obesity, T2DM), which suggests protective activities against those diseases [208]. In addition, FGF21 could be secreted from the brown adipose tissue and participate in thermoregulation in an ATF4-dependent way [209]. Several FGF21 analogues have been clinically tested as promising NAFLD treatments.

A long-acting FGF21 analogue, PF-05231023, had been tested on obese patients with and without T2DM. PF-05231023 significantly reduced TG levels, and increased HDL-C and adiponectin [210]. Another PEGylated FGF21 analogue, pegbelfermin, has been tested in a phase 2a study on NAFLD patients with obesity. In that study, 16 weeks of subcutaneous pegbelfermin administration resulted in a significant reduction of liver fat [211]. AKR-001 (FGF21 analogue) has a positive influence on lipoprotein profile (TG, nHDL-C, HDL-C, APOB, and APOC3) and improved insulin sensitivity [212,213]. Because FGF21 is quickly deactivated by proteolysis, some attempts have been made to prevent FGF21 cleavage by inhibiting its main protease FAP (seprase). BR103354, a FAP inhibitor, was tested in vitro, in mice and non-human primate models, where it was shown to reduce non-fasting glucose and TG levels, and improve hepatic steatosis and fibrosis. This suggests FAP inhibitors as potential anti-diabetic and anti-NASH medications [214].

The anti-atherosclerotic effect of FGF21 was studied in several clinical trials, where it was shown to significantly improve the cardiometabolic profile in obese patients with T2DM [215]. FGF21 therapy significantly improves lipid profiles, reduces vascular inflammation, and mitigates apoptosis and OS in atherosclerosis-related diseases [216].

Despite multiple positive outcomes in reports regarding safety and good tolerability of long-term FGF21 application, there are also several known drawbacks. As it was found in mice, FGF21 inhibits osteoblastogenesis via PPARγ, thus connecting bone turnover and energy metabolism [217]. Similar effects on the bone-turnover markers were also found n T2DM human patients in a trial for FGF21 analogue PF-05231023, where N-terminal propeptides and C-telopeptide cross-linking of type 1 collage were altered [210]. Further, depending on dose, up to 92% of subjects had increased titre of the anti-FGF21 antibodies, which raise the concern of immunogenicity in the case of long-term NAFLD treatment with FGF21 and its analogues [218]. However, it is necessary to note that despite the positive effect of FGF21 and its analogues on metabolic comorbidities of NAFLD and reduced liver fat, a recent systematic study suggests that better outcomes could be achieved with weight loss via diet modification and exercise [219].

In total, we could find a promising trend in developing NAFLD treatment. Currently, several medications able to treat NAFLD and provide CVD protection are in different stages of clinical trials. However, keeping in mind the defined drawbacks of the FGF21-based drugs, the efficacy of these therapies should be defined in long-term studies and in patients with severe NASH and a high risk of CVD.

## 5. Conclusions

The close association between NAFLD and CVD is supported by observations that CVD is the most common cause of death among NAFLD patients. Liver mitochondrial fatty-acid β-oxidation is the primary system that reacts to disbalances in nutrient flow. Subsequent alterations in the liver lipid metabolism drive NAFLD development, simultaneously creating a CVD-favouring pro-atherogenic environment via system-wide CK production, dyslipidemia, IR, and procoagulant imbalance. NAFLD development and lipid-profile alterations are regulated by a complex network of genes reacting to intracellular and environmental stresses, circadian rhythms, nutrients, and lifestyle. Currently, several promising NAFLD/NASH therapies with CVD-protecting activities are in development. However, given the extremely diverse nature of metabolic pathways involved in genetic NAFLD pathogenesis, a clear understanding of the underlying molecular mechanisms is required to provide effective care and treatment. Despite significant success in the understanding of NAFLD–CVD causality and a wide range of available pharmacological tools, great efforts should be oriented on the promotion of a healthy lifestyle, nutrition literacy, and smoking cessation, thus contributing to the prevention of the primary causes of NAFLD and CVD.

## Figures and Tables

**Table 1:** Recent results obtained in mouse models and elucidating effects of different genes on molecular aspects of NAFLD pathogenesis and general lipid metabolism.

Gene/Target	Mutant/Line	Results	Reference
Mitochondrial Functions			
*Ech1* (Enoyl coenzyme A hydratase 1)	Ech1 OE; C57BL/6	Ech1 OE ameliorates lipid accumulation, liver injury, dyslipidemia, and IR.	[115]
*LPGAT1* (Lysophosphatidylglycerol Acyltransferase 1)	LPGAT1^−/−^; C57BL/6	LPGAT1 deficiency protected mice from diet-induced obesity, but led to hepatopathy, insulin resistance, and NAFLD as a consequence of OS, mitochondrial DNA depletion, and mitochondrial dysfunction.	[116]
*CRLS1* (Cardiolipin synthase 1)	CRLS1^−/−^; C57BL/6	Crls deficiency resulted in a prominently aggravated lipid metabolism disorder, inflammation, and fibrosis; CRLS1 suppressed ATF3 expression and inhibits its activity in palmitic-acid-stimulated hepatocytes	[117]
*MFN2* (Mitofusin 2)	Liver-specific Mfn2 KO mice; C57BL/6J	Mouse NASH models present lower levels of Mfn2 in the liver, and the re-expression of Mfn2 in the liver ameliorates the NASH phenotype. Hepatic Mfn2 ablation causes a NASH-like phenotype that progresses to liver cancer with age. Mfn2 binds to and participates in the transfer of PS. Hepatic Mfn2 deficiency causes a reduced transfer of PS from ER to mitochondria, which leads to reduced PS synthesis and ER stress, in turn causing inflammation, fibrosis, and liver cancer.	[118]
Mitochondrial *GNMT* (glycine N-methyltransferase)-Complex II	Liver-specific repression of the GNMT by miR-873-5p; C57BL/6	GNMT expression is controlled by miR-873-5p in the hepatocytes, leading to disruptions in mitochondrial functionality. NASH therapies based on anti-miR-873-5p resolve lipid accumulation, inflammation, and fibrosis by enhancing fatty-acid β-oxidation in the mitochondria.	[119]
mTORC1	Diet with high/low ratio of ω-3/ω-6 polyunsaturated fatty acids; C57BL/6	Body weight, atherosclerosis marker, insulin signal index, and level of lipid accumulation in the liver were significantly lowered in the high group. Expressions of p-mTOR and raptor were inhibited by high ω-3 PUFAs. High ω-3 PUFAs depressed p-mTOR and raptor expressions, regulated ETC and TCA cycle pathway, and increased activities of mitochondrial complexes I, II, III, IV, and V.	[120]
*GADD45GIP1* (*CRIF1*); *GDF15* and *FGF21*	Liver-specific Crif1-deleted mice; GDF15 and FGF21 null mice; C57BL/6J	Crif1 KO mice showed lower hepatic lipid accumulation, which was associated with lower hepatic expression of Srebp1, Srebp1c, and Cd36; Crif1 KO mice were resistant to diet-induced obesity and protected against hepatic steatosis and insulin resistance when fed an HFD.	[121]
*RAB24*	Human-delivered samples; Hepa1-6 cells; FGF21 and RAB24 KO mice; C57BL/6N	Rab24 directly interacts with FIS1, thus regulating mitochondrial turnover. Reduction of Rab24 causes reduced mitochondrial fission resulting in enhanced energy usage. Rab24 KO reassembles the fasting state, whereby mitochondria are metabolically reprogrammed towards higher respiration through enhanced connectivity and bioenergetic efficiency.	[122]
*MCJ* (Methylation-controlled J protein)	Leptin receptor mutant (Leprdb/J); C57BL/6J	The therapeutic inhibition of MCJ expression in vivo enhances FAO in the liver in a NASH model. The enhanced FAO resulting from inhibiting MCJ is due to enhanced Complex I activity. In vivo treatment of siMCJ of mice with NASH increases β-oxidation and decreases lipid accumulation in the liver, but does not increase ROS production.	[123]
*ANT2* (ADP/ATP translocase 2)	Liver-specific Ant2 cKO mice; ANT2 inhibition by carboxyatractyloside (CATR)	Targeted disruption of Ant2 in mouse liver enhances uncoupled respiration without damaging mitochondrial integrity and liver functions. Liver-specific Ant2 KO mice are leaner and resistant to hepatic steatosis, obesity and insulin resistance under a lipogenic diet.	[124]
*GRK2* (G protein-coupled receptor kinase 2)	GRK2 hemizygous mice	GRK2± mice were protected from HFD-induced NAFLD. GRK2± mice preserved hepatic protective mechanisms as enhanced autophagy and mitochondrial fusion and biogenesis, together with reduced endoplasmic reticulum stress. Enhanced GRK2 expression potentiated palmitic-acid-triggered lipid accumulation in human hepatocytes directly relating GRK2 levels to steatosis.	[125]
AMPK-CPT Signalling Pathway			
*ACC1*; *ACC2* (Acetyl-CoA carboxylase)	KKAy or C57BL/6J mice; ACC1 and ACC2 liver-specific KO	Deletion of ACCs decreased PUFA concentrations in the liver due to reduced malonyl-CoA. PUFA deficiency induced SREBP-1c, which increased GPAT1 expression and VLDL secretion. Thus, inhibiting lipogenesis in humans reduced hepatic steatosis, but inhibiting ACC resulted in hypertriglyceridemia due to activation of SREBP-1c and increased VLDL secretion.	[126]
*ACC2*	ACC2 KO mice; C57BL/6J	The global deletion of ACC2 enhances lipid disposal without competing with glucose metabolism at the whole-body and skeletal-muscle levels. This successful lipid reduction is characterized by a decreased acetyl-CoA pool in skeletal muscle, which is accounted for by enhanced TCA cycle activity and acetyl-CoA conversion into acetylcarnitine.	[127]
*ROCK1* (Rho-kinase 1)	Liver-specific ROCK1 deletion; *C57BL/6J*	Mice lacking ROCK1 in the liver were resistant to diet-induced obesity owing to increased energy expenditure and thermogenic gene expression. Treatment with metformin reduced hepatic lipid accumulation by inactivating ROCK1, resulting in activation of AMPK downstream signalling.	[128]
*SIRT1* (Sirtuin 1), *AMPKα* (AMP-activated protein kinase); AITC (Allyl isothiocyanate) treatment	Sirt1 and AMPKα; AML-12 cells; C57BL/6	AITC attenuates inflammation by inhibiting the NF-κB signalling pathway in vitro and de novo lipogenesis, and promotes FAO by activating the Sirt1/AMPK signalling pathway in vitro.	[129]
*SIRT5*; *LCAD* (Long-chain acyl-CoA dehydrogenase)	SIRT5 and LCAD knockout C57BL/6	Medium-chain triglycerides (MCT), containing C8–C12 FA degradation, was significantly reduced in the Sirt5KO liver. This decrease was localized to the mitochondrial β-oxidation pathway, as Sirt5KO mice exhibited no change in peroxisomal C12 β-oxidation. ER ω-oxidation was increased in Sirt5KO liver. LCAD KO mice developed periportal macrovesicular steatosis when fed coconut oil.	[130]
Adiponectin-based agonist JT003	HepG2 and human hepatic activated stellate cell line LX2; C57BL/6J	AdipoRs dual agonist JT003 with a longer half-life could ameliorate NASH and related liver fibrosis via AMPK, PPARα, and PI3K-Akt signal pathways. JT003 treatment significantly improves the function of the ER–mitochondrial axis, which contributes to the reduced HSC activation.	[131]
*GGPPS* (Geranylgeranyl pyrophosphate synthase)	Liver-specific GGPPS deletion; *C57BL/6J*	Long-term HFD decreases GGPPS expression, which shifts the fuel preference from FAs toward glucose. Liver-specific Ggpps deficiency drives the Warburg effect by impairing mitochondria function, and induces hepatic inflammation. Ggpps deficiency enhances the hyper-farnesylation of liver kinase B1 and promotes metabolic reprogramming by regulating AMPK activity.	[132]
Mouse *CREBH* (CAMP-responsive element-binding protein, hepatic-specific) site-directed mutagenesis, transfection (OE)	Mouse AML-12 cells, human hepatocyte: HepG2 and HEK293T cells; C57BL/6J	N-glycosylation of CREBH modulated the production of PPARα and activation of SCD-1 by interfering with the recognition of CRE in their promoters, inducing CREBH/PPARα and CREBH/SCD-1 interaction. This subsequently improved the synthesis of hepatic lipids and sterols and relieved inflammation, lipotoxicity, and lipid peroxidation.	[133]
*IMP2* (Insulin-like growth factor 2 MRNA binding Protein 2)	Hepatocyte-specific IMP2 knockout; C57BL/6	IMP2 binds and stabilizes the mRNAs encoding the critical regulators of hepatic fatty-acid oxidation, PPARα and CPT-1A; loss of IMP2 diminishes the abundance of those mRNAs, resulting in reduced mitochondrial fatty-acid oxidation. Mice with hepatic IMP2 deficiency fed an HFD show a modest, progressive accumulation of hepatic triglycerides beyond that of HFD-fed controls, ultimately reflected in elevated circulating triglycerides and mildly elevated blood glucose.	[134]
FOH (Farnesol)	Steatotic HepaRG cells	FOH treatment increases FAO and decreases TG accumulation in steatotic HepaRG cells, which is likely attributable to PPARα-mediated induction of mitochondrial FAO.	[135]
*TFF3* (Trefoil factor 3)	TFF3 KO; C57BL/6	Tff3 binds the promoter of PPAR and up-regulates hepatic FAO.	[136]
*CPT1A* (Human carnitine palmitoyltransferase 1A)	C57BL/6	Expression of hCPT1AM (a mutated isoform that is insensitive to malonyl-CoA) enhanced hepatic FAO and autophagy, reduced liver steatosis, and improved glucose homeostasis.	[137]
*CPT2* (Carnitine palmitoyltransferase 2)	Liver-specific deficiency of CPT2; C57BL/6	Cpt2^L−/−^ mice were resistant to HFD-induced obesity and glucose intolerance with an absence of liver damage, although they exhibited serum dyslipidemia, hepatic oxidative stress, and systemic carnitine deficiency. Feeding an HFD induced hepatokines in mice, with a loss of hepatic fatty-acid oxidation that enhanced systemic energy expenditure and suppressed adiposity.	[73]
Antioxidant			
*SOD1* (Cu/Zn-superoxide dismutase)	Sod1^−/−^; C57BL/6	Excess fat accumulation in the livers of Sod1KO mice due to impaired VLDL secretion leads to NAFLD, and the high OS triggers necroptosis in the liver, leading to the generation of DAMPs. The DAMPs activate macrophages and the inflammasome leading to the production of pro-inflammatory cytokines, resulting in non-resolving chronic inflammation.	[138]
*PRX5* (Peroxiredoxin)	PRX5 *KO*; C57BL/6J; HepG2 cells	Prx5 ameliorated FFA-induced ROS overproduction and lipid accumulation in HepG2 cells. Prx5 overexpression ameliorated hepatic steatosis by regulating lipogenesis and hepatic inflammation. Additionally, upon NAFLD induction, the expression of lipogenesis-related proteins increased more among Prx5 KO mice than among WT mice.	[139]
*CAT* (catalase)	CAT knockout C57BL/6; HepG2 cells;	The fat accumulation, lipid peroxidation, and H_2_O_2_ release were significantly elevated in HFD CAT KO mice. The liver mitochondria tended to be more severely damaged, and mitochondrial DNA copy number and cellular ATP concentrations were significantly lower in CAT KO mice. In CAT KO HepG2 cells, fatty-acid treatment causes accelerated cellular lipid accumulation and depressed mitochondrial biogenesis.	[140]
General Lipid Metabolism			
*GCN2* (General control nonderepressible 2)	Gcn2^−/−^; H9C2 cells; C57BL/6	Gcn2^−/−^ significantly attenuated HFD-induced liver dysfunction, hepatic steatosis, and insulin resistance; Exercised GCN2-deficient mice have enhanced efficacy in improving hepatic steatosis and liver lipid metabolism, at least partially, via the AMPK/SIRT1/PPARα pathway. GCN2 deficiency protects cardiac function by reducing lipid accumulation, OS, and cell death by inhibiting eIF2α -ATF4-CHOP signalling.	[141,142,143,144]
*FABP1* (Fatty-acid-binding protein 1)	FABP1 OE; C57BL/6	Exercise down-regulated the FABP1 signalling pathway, which was most closely associated with lipid metabolism. Liver-specific overexpression of FABP1 abolished the protective effect of exercise in NAFLD mice. Exercise significantly increased autophagic flux via restoring lysosomal function, including lysosomal proteolysis and lysosomal acidification maintenance, contributing to enhancement in autophagic clearance and subsequent alleviation of hepatic steatosis.	[145]
*APOE* (Apolipoprotein E) and *RON* (Macrophage stimulating 1 receptor)	ApoE^−/−^/Ron^−/−^; C57BL/6	Double KO mice had features of steatosis, inflammation, OS, and hepatocyte damage, as well as increased accumulation of FAs in the liver and decreased levels of bile acids.	[146]
*STING* (Tmem173)	STING^−/−^; C57BL/6	STING deficiency attenuated steatosis, fibrosis, and inflammation; increased fasting glucose levels in mice independently of insulin; reduced levels of cholesterol, triglycerides, and LDL in serum; enhanced levels of HDL; reduced levels of mtDNA in hepatocytes, TNF-α, and IL-6 expression in cultured Kupffer cells; and reduced mRNA levels of Col1A1 and α -SMA in livers.	[147]
*SPP1* (Osteopontin)	Spp1^−/−^; C57BL/6	Spp1^−/−^ mice had increased lipid accumulation, high levels of ALT, fatty-acid translocase (CD36/FAT), pro-fibrogenic markers (Col1a1, Col 4a1, Timp1), and insulin secretion; while hepatic FOXO1 was downregulated.	[148]
*LRP1* (LDL receptor-related protein-1)	LRP1 with distal NPxY motif mutation; C57BL/6	Dysfunction of LRP1 is protective against HFHC diet-induced dyslipidemia, fatty liver disease, and neuroinflammation.	[149]
*TM6SF2* (Transmembrane 6 superfamily member 2)	Tm6sf2^−/−^; C57BL/6	APOB and ER lipid raft protein (ERLIN) 1 and 2 were TM6SF2-interacting proteins. ERLINs and TM6SF2 mutually bound and stabilized each other. TM6SF2 bound and stabilized APOB via two luminal loops. ERLINs did not interact with APOB directly, but still increased APOB stability through stabilizing TM6SF2. Defective APOB stabilization, as a result of ERLIN or TM6SF2 deficiency or E167K mutation, is a key factor contributing to NAFLD.	[150]
*LAMP2A* (Lysosome-associated membrane protein 2A)	LAMP2A^fl/fl^ Cre+; C57BL/6	LAMP2A reduction resulted in decreased levels of (chaperone-mediated autophagy) CMA-positive regulators. Deleting LAMP2A hindered lipid droplet (LD) breakdown, but not LD formation. The disruption of CMA-induced perilipin 5 (Plin5) degradation was an obstacle to LD breakdown, explaining the lipid homeostasis imbalance in NAFLD.	[151]
*LCHAD* (Long-chain 3-hydoxyacyl-CoA dehydrogenase)	LCHAD heterozygous mice	LCHAD mice developed significant hepatic steatosis starting at a young age (3 months old) and HCC at an older age (>13 months old) without any evidence of fibrosis or cirrhosis. LCHAD defects predispose to HCC, and mitochondrial dysfunction plays an important role in HCC pathogenesis.	[152]
*CLOCK* (Circadian locomotor output cycles kaput) and *APOE* (Apolipoprotein E)	Clk^Δ19/Δ19^, Apoe^−/−^; C57BL/6	CLOCK regulates HIF1α protein levels by binding to the E-boxes in the promoters and modulating the expression of PHD proteins that regulate HIF1α protein stability. HIF1α binds to the Cd36 promoter to increase the expression of CD36 and uptake of fatty acids by the liver. Thus, a regulatory mechanism involving circadian CLOCK, hypoxia signalling, and lipid metabolism protects against NAFLD.	[153]
*SLUG* (Snail family transcriptional repressor 2)	Slug^Δhep^; C57BL/6	Slug is a new lipogenic TF that promotes de novo lipogenesis by an epigenetic mechanism. Hepatocyte-specific deletion of Slug inhibited the hepatic lipogenic program and protected against obesity-associated NAFLD, IR, and glucose intolerance; Slug-associated Lsd1 mediates lipogenesis by demethylating H3K9 on the Fasn promoter, suggesting a new demethylation lipogenic insulin/Slug/Lsd1/H3K9 pathway that promotes NAFLD and T2DM.	[154]
*LAP1* (Lamina-associated polypeptide 1) and *TOR1A* (TorsinA), an AAA+ ATPase	Lap1^fl/fl^ and Tor1a^fl/fl^; C57BL/6	The torsinA/LAP1 pathway regulates VLDL secretion and liver fat accumulation. Conditional deletion of either LAP1 or torsinA from hepatocytes caused profound steatosis.	[155]
*XBP1* (Xbp1-X-box binding protein 1)	AlbCre;Xbp1^flx/flx^; C57BL/6	XBP1 is a 12 h clock manner that regulates gene expression, cellular membrane fluidity, and mitochondrial utilization of fatty-acid and glucose substrates; XBP1 provides temporal transcriptional regulation of the key metabolic enzymes such as SCD1, LPCAT3, and LCAT.	[156]
*TBK1* (TANK-binding kinase 1)	Liver-specific TBK1 knockout; C57BL/6J	TBK1 impacts lipid metabolism via binding to the key rate-limiting enzyme ACSL1. In the fasted state, TBK1 expression is induced, but remains inactive, and can serve as a molecular scaffold to localize ACSL1 to the mitochondrial outer membrane, thus facilitating fatty-acid β-oxidation. In the absence of TBK1, fasting-stimulated ACSL1 localization to mitochondria is blunted, driving the localization of the enzyme to the ER for fatty-acid re-esterification.	[157]
Supplementation			
Fisetin injection	FL83B cells; C57BL/6	Fisetin treatment had decreased body weight and epididymal adipose tissue weight; reduced liver LD and hepatocyte steatosis, and alleviated serum FFA and leptin concentrations; significantly decreased FAS; and significantly increased phosphorylation of AMPKα and the production of sirt-1 and CPT1 in the liver tissue. In vitro, fisetin decreased lipid accumulation and increased lipolysis and β-oxidation in hepatocytes.	[158]
Phloretin supplementation/treatment	HepG2 cells; C57BL/6	Phloretin significantly reduced excessive lipid accumulation and decreased SREBP-1c, blocking the expression of FAS in oleic acid-induced HepG2 cells. Phloretin increased Sirt1 and phosphorylation of AMPK to suppress ACC expression, reducing FA synthesis in hepatocytes.	[159]
Chitosan oligosaccharide (ChO) treatment	Inflammation and OS; C57BL/6	ChO treatment decreases the serum levels of AST and ALT, Il-6, Il-1β, Tnf-α, and lowers lipid accumulation; and induces higher expression of fatty β-oxidation-related genes PPARα and *CPT1*, and OS-related genes (*NQO1*, *HO*-1, *GSTA1*.	[160]
HC (High cholesterol) diet	Mitochondrial parameters; C57BL/6	HC-diet-induced liver damage and dysfunction, associated with a decrease in mitochondrial membrane potential and ATP production, increased cholesterol levels in the organelle. Mitochondria adapt to high levels of cholesterol content, increasing fission and decreasing apoptosis, while damaged hepatocytes do not enter apoptosis and proliferate, perpetuating liver damage.	[161]
A mitochondria-targeted fatty acid analogue 1-triple TTA	Hepatic glucose homeostasis; Wistar rats	The mitochondrially targeted fatty-acid analogue 1-triple TTA seemed to lower hepatic glucose and glycogen levels by inhibition of gluconeogenesis. This was also linked to a reduction in glucose oxidation accompanied by reduced pyruvate dehydrogenase activity and stimulation of ME1 and G6PD, favouring a shift from glucose to FAO.	[162]
Glucose and fructose supplementation	Hepatic glucose homeostasis; C57BL/6	Glucose and fructose increased ChREBP-β levels, and fructose supplementation uniquely increased SREBP1c and downstream fatty-acid-synthesis genes, resulting in reduced liver insulin signalling. In contrast, glucose enhanced total ChREBP expression and triglyceride synthesis, but was associated with improved hepatic insulin signalling.	[84]
Feeding with 1.5X branched-chain amino acids (BCAAs)	Ketogenesis and hepatic mitochondrial oxidation; C57BL/6	Long-term exposure of the mice to the BCAA-modified diet resulted in a chronic ketogenic environment. Metabolic profiling demonstrated that chronic induction of the hepatic mitochondrial oxidative networks (β-oxidation, ketogenesis, TCA cycle) occurred together with lower rates of lipogenesis in the liver of the ketogenic mice.	[163]
Recombinant human relaxin-2 supplementation	C57BL/6	Human relaxin-2 attenuated steatosis and increased phosphorylation of IRS1, Akt eNOS, and activated genes that regulate fatty-acid oxidation and suppressed ACC.	[164]
Fructose supplementation	Hepatic FAO; C57BL/6	Fructose supplementation increased fatty-acid synthesis mediated via upregulation of SREBP1c and ChREBP-β, in which glucose supplementation increased TG synthesis associated with upregulation of ChREBP. Fructose increased hepatic malonyl-CoA levels and increased acetylation of ACADL and CPT1a, while glucose supplementation resulted in increased acetylation of HADA/B.	[90]
Supplementation with SFA (saturated fatty acids)	SFA-induced lipotoxicity; C57BL/6	High-level palmitate (HPA) induces lipotoxic effects in liver cells, while low-level PA (LPA) increases mitochondrial functions and alleviates the injuries induced by HPA or by hepatotoxic agent CCl4 (carbon tetrachloride). LPA-mediated mitochondrial homeostasis is regulated by CDK1-mediated SIRT3 phosphorylation, which in turn deacetylates and dimerizes CPT2 to enhance FAO.	[165]
Other			
*CES1* (Carboxylesterase 1)	Ces1^−/−^; C57BL/6	Ces1d-deficient mice were protected from HSD-induced hepatic lipid accumulation. Ces1d deficiency leads to activation of AMPK and inhibitory phosphorylation of ACC.	[166]
*VSIG4* (V-set and immunoglobulin domain-containing protein-4)	Vsig4^−/−^; C57BL/6	Loss of Vsig4 accelerated the severity of lipid deposition, fibrosis, and the inflammatory response via the NF-kB and TGFβ 1 signalling pathways.	[167]
*CCN1* (Cellular communication network factor 1)	C57BL/6	CCN1 OE up-regulates the expression of fatty-acid metabolism-associated genes; it increased the expression of cleaved caspase 3 and the pro-apoptotic protein Bax.	[168]
*DPP4* (Dipeptidyl peptidase-4)	DPP4 inhibitor (DPP4i); C57BL/6J; HepG2 cell line	DPP4i administration reduced serum liver enzyme and hepatic triglyceride levels and markedly improved hepatic steatosis and fibrosis in the AMLN-diet-induced NASH model. DPP4i may efficiently attenuate the pathogenesis of AMLN diet-induced NASH in mice by suppressing lipotoxicity-induced apoptosis.	[169]
*STK25* (Serine/threonine kinase 25)	Liver-specific triantennary GalNAc-conjugated ASO targeting *STK 25*; *C57BL/6J*	Hepatocyte-targeting GalNAc-*Stk25* ASO in obese mice effectively ameliorated steatosis, inflammatory infiltration, hepatic stellate-cell activation, nutritional fibrosis, and hepatocellular damage in the liver, without any systemic toxicity or local tolerability concerns. Also, treated mice were protected against HF-diet-induced hepatic oxidative stress and had improved mitochondrial function.	[170]
*SMOC2* (Secreted modular calcium-binding protein 2)	SMOC2 KO; C57BL/6	SMOC2 expression promoted hepatic steatosis by interacting with TGF-β 1 to regulate lipid metabolism, fibrosis, and inflammation.	[171]
*ELAVL1* (RNA-binding protein HuR)	Hepatocyte-specific HuR knockout; C57BL/6	HuR controls the production of CYCS, NDUFB6, UQCRB, and APOB; preserves the ability of mitochondria to produce energy; and maintains lipid homeostasis.	[172]
*FNDC5* (Fibronectin type III domain-containing protein 5)	C57BL/6; mouse primary hepatocytes and HepG2 cells	FNDC5 regulates liver steatosis, and insulin resistance and injury, thus limiting the development and progression of NAFLD; irisin promotes mitochondrial biogenesis and reduces OS.	[173]
*S100A11* (S100 calcium-binding protein A11)	Ghr-floxed x(B6; FVB-Tg (adipoq-Cre)1Evdr/J)	A high-fat diet promotes liver S100A11 expression, which may interact with HDAC6 to block its binding to FOXO1, releasing or increasing the acetylation of FOXO1, thus activating autophagy and lipogenesis, and accelerating lipid accumulation and liver steatosis.	[174]
*CASP1* (Caspase-1), *CASP11*, *PR3* (Prtn3), and *NE* (Elane)	Casp1^−/−^/Casp11^−/−^/NE^−/−^/PR3^−/−^; C57BL/6	Mutants were protected from developing diet-induced weight gain, liver steatosis, and adipose tissue inflammation	[175]
*ClC-2* (Chloride voltage-gated channel 2)	Chloride channel 2 KO mice; C57BL/6; HepG2 cells	Knockdown of ClC-2 in liver-attenuated HFD-induced weight gain, obesity, hepatocellular ballooning, and liver lipid accumulation and fibrosis, accompanied by reduced plasma FFA, TG, TC, ALT, AST, glucose, and insulin levels. HFD-fed ClC-2 KO mice showed inhibited hepatic lipid accumulation via regulating lipid metabolism through decreasing SREBP-1c expression and its downstream targets (such as FAS, HMGCR, and ACCα).	[176]
*ABCB10* (ATP-binding cassette sub-family B member 10, mitochondrial)	Liver-specific deficiency of ABCB10; C57BL/6	ABCB10 was identified as a mitochondrial biliverdin exporter. Diet-induced obese mice with liver-specific ABCB10 deletion were protected from steatosis and hyperglycemia, and had improved insulin-mediated suppression of glucose production and decreased expression of lipogenic SREBP-1c. Protection was concurrent with enhanced mitochondrial function and increased inactivation of PTP1B (protein tyrosine phosphatase non-receptor type 1), a phosphatase that disrupts insulin signalling and elevates SREBP1c expression. Thus, as a lipophilic hydrogen peroxide scavenger, bilirubin was the maladaptive effector linked to ABCB10 function.	[177]

## Data Availability

Not applicable.

## Abbreviations

ACAA2	acetyl-CoA acyltransferase 2
ACAD	acyl-CoA dehydrogenase
ACC	acetyl-CoA carboxylase
AITC	allyl isothiocyanate
AMPK	AMP-activated protein kinase
ANT2	ADP/ATP translocase 2
APOE	apolipoprotein E
ATF4	activating transcription factor 4
BAT	brown adipose tissue
CACT	acylcarnitine translocase
CASP1	caspase-1
CAT	catalase
CCl4	carbon tetrachloride
CCN1	cellular communication network factor 1
CES1	carboxylesterase 1
ChO	chitosan oligosaccharide
CHOP	C/EBP homologous protein
ChREBP	carbohydrate-responsive element-binding protein
ClC-2	chloride voltage-gated channel 2
CLOCK	circadian locomotor output cycles kaput
CPT	carnitine palmitoyltransferase
CREBH	CAMP-responsive element-binding protein, hepatic-specific
CRLS1	cardiolipin synthase 1
CVD	Cardiovascular diseases
DAGs	diacylglycerols
DNL	de novo lipogenesis
DPP4	dipeptidyl peptidase-4
ECHS1	enoyl-CoA hydratase, short chain 1
ELAVL1	RNA-binding protein HuR
ERK1/2	extracellular signal-regulated kinases 1 and 2
ETC	electron transport chain
FA	fatty acid
FABP1	fatty-acid-binding protein 1
FACS	fatty-acid synthase
FAT	fatty-acid translocase
FGF21	fibroblast growth factor 21
FGFR1	FGF receptor 1
FNDC5	fibronectin type III domain-containing protein 5
FOH	farnesol
FRS2α	fibroblast growth factor receptor substrate 2 alpha
GCN2	general control nonderepressible 2
GDF15	growth/differentiation factor 15
GGPPS	geranylgeranyl pyrophosphate synthase
GNMT	glycine N-methyltransferase
GRK2	G protein-coupled receptor kinase 2
HADH	hydroxyacyl-CoA dehydrogenase
HC	high cholesterol
HCC	hepatocellular carcinoma
HDL-C	high-density lipoprotein cholesterol
IL-6	Interleukin 6
IMP2	insulin-like growth factor 2 mRNA binding protein 2
IGFBP1	Insulin-like growth factor-binding protein 1
IR	insulin resistance
IRS1	insulin receptor substrate 1
JNK	c-Jun N-terminal kinase
KLB	β-Klotho
LAMP2A	lysosome-associated membrane protein 2A
LAP1	lamina-associated polypeptide 1
LCAD	long-chain acyl-CoA dehydrogenase
LCFAs	long-chain FAs
LCHAD	long-chain 3-hydoxyacyl-CoA dehydrogenase
LDH	lactate dehydrogenase
LDL	low-density lipoprotein
LKB1	liver kinase B1
LPGAT1	lysophosphatidylglycerol acyltransferase 1
LRP1	LDL receptor-related protein-1
LXR	liver X receptor
MCD	malonyl-CoA decarboxylase
MCJ	methylation-controlled J protein
MFN2	mitofusin 2
MetS	metabolic syndrome
MPC	mitochondrial pyruvate carrier 1
MUPs	major urinary proteins
NAFLD	non-alcoholic fatty liver disease
NASH	non-alcoholic steatohepatitis
NF-kB	nuclear factor kappa B
NLRP3	NLR family pyrin domain-containing 3
OS	oxidative stress
PA	palmitate
Plin5	perilipin 5
PCs	phosphatidylcholines
PDH	pyruvate dehydrogenase
PDK	pyruvate dehydrogenase kinase
PDP	pyruvate dehydrogenase phosphatase
PRX5	peroxiredoxin
PTP1B	protein tyrosine phosphatase non-receptor type 1
ROCK1	rho-kinase 1
RON	macrophage-stimulating 1 receptor
S100A11	S100 calcium-binding protein A11
SFA	saturated fatty acids
SIRT1	sirtuin 1
SLUG	snail family transcriptional repressor 2
SMOC2	secreted modular calcium-binding protein 2
SOD1	Cu/Zn-superoxide dismutase
SREBP1c	sterol regulatory element-binding protein-1c
STK25	serine/threonine kinase 25
T2DM	type 2 diabetes mellitus
TBK1	TANK-binding kinase 1
TC	total cholesterol
TCA cycle	tricarboxylic acid cycle
TGF-β	transforming growth factor beta
TFF3	trefoil factor 3
TM6SF2	transmembrane 6 superfamily member 2
TNF-α	tumor necrosis factor alpha
UCP2	uncoupling protein 2
VSIG4	V-set and immunoglobulin domain-containing protein-4
XBP1	Xbp1-X-box binding protein 1
